# Do second generation sequencing techniques identify documented genetic markers for neonatal diabetes mellitus?

**DOI:** 10.1016/j.heliyon.2021.e07903

**Published:** 2021-08-30

**Authors:** Imran Ali Khan

**Affiliations:** Department of Clinical Laboratory Sciences, College of Applied Medical Sciences, King Saud University, PO Box-10219, Riyadh, 11433, Saudi Arabia

**Keywords:** Neonatal diabetes mellitus (NDM), First-generation sequencing, DNA-Sanger sequencing, Second-generation sequencing, Next-generation sequencing (NGS), Exome sequencing (ES), Whole exome sequencing (WES), Whole genome sequencing (WGS), Targeted gene panels

## Abstract

Neonatal diabetes mellitus (NDM) is noted as a genetic, heterogeneous, and rare disease in infants. NDM occurs due to a single-gene mutation in neonates. A common source for developing NDM in an infant is the existence of mutations/variants in the *KCNJ11* and *ABCC8* genes, encoding the subunits of the voltage-dependent potassium channel. Both *KCNJ11* and *ABCC8* genes are useful in diagnosing monogenic diabetes during infancy. Genetic analysis was previously performed using first-generation sequencing techniques, such as DNA-Sanger sequencing, which uses chain-terminating inhibitors. Sanger sequencing has certain limitations; it can screen a limited region of exons in one gene, but it cannot screen large regions of the human genome. In the last decade, first generation sequencing techniques have been replaced with second-generation sequencing techniques, such as next-generation sequencing (NGS), which sequences nucleic-acids more rapidly and economically than Sanger sequencing. NGS applications are involved in whole exome sequencing (WES), whole genome sequencing (WGS), and targeted gene panels. WES characterizes a substantial breakthrough in human genetics. Genetic testing for custom genes allows the screening of the complete gene, including introns and exons. The aim of this review was to confirm if the 22 genetic variations previously documented to cause NDM by Sanger sequencing could be detected using second generation sequencing techniques. The author has cross-checked global studies performed in NDM using NGS, ES/WES, WGS, and targeted gene panels as second-generation sequencing techniques; WES confirmed the similar variants, which have been previously documented with Sanger sequencing. WES is documented as a powerful tool and WGS as the most comprehensive test for verified the documented variants, as well as novel enhancers. This review recommends for the future studies should be performed with second generation sequencing techniques to identify the verified 22 genetic and novel variants by screening in NDM (PNDM or TNMD) children.

## 1 Introduction

**Figure 1:**
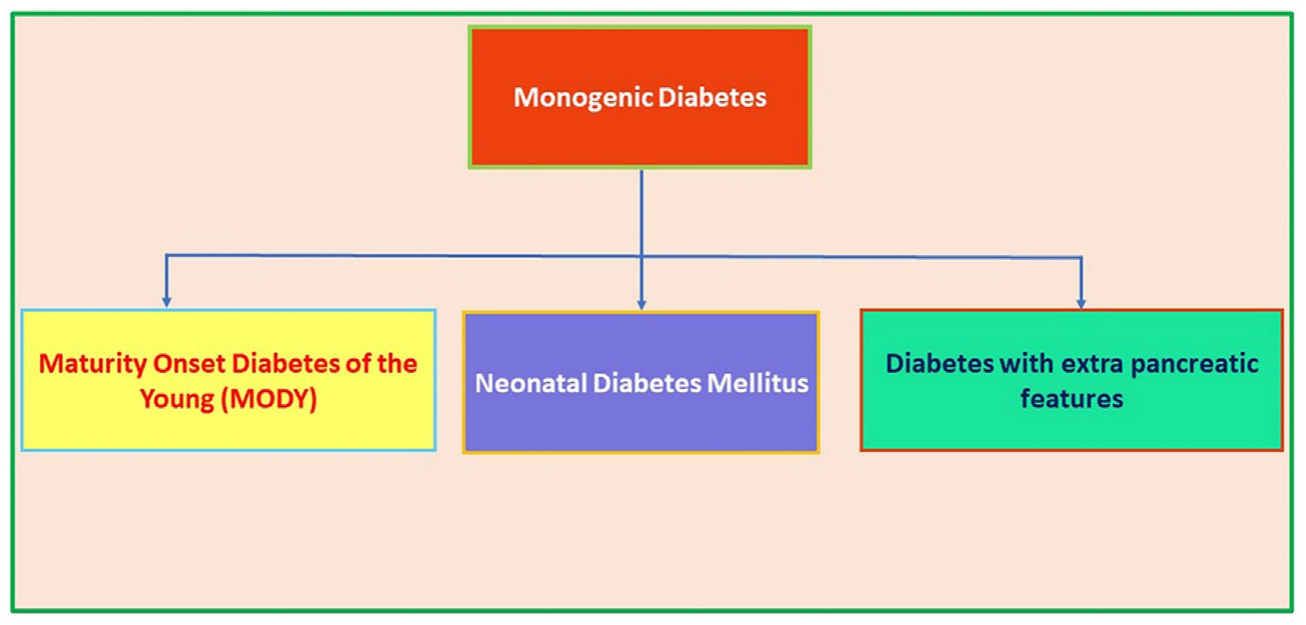
Different modes of occurrence of early-onset diabetes.

Diabetes Mellitus is a group of metabolic disorders with various etiologies that are defined by elevated serum glucose levels or persistent hyperglycemia, resulting in abnormalities of insulin secretion, insulin action, or both. Diabetes is classified as monogenic and polygenic diabetes. Monogenic diabetes is caused by a single diabetic gene defect that is usually inherited as a dominant or recessive trait which is also caused due to rare forms of non-autoimmune diabetes that contribute to the disease phenotype affecting normal pancreatic β-cell physiology, development, and differentiation. Monogenic diseases in families can be inherited as dominant, recessive, non-Mendelian trait or can occur as the result of a mutation from *de novo*, i.e. not inherited from parents. Neonatal diabetes mellitus (NDM) or Congenital diabetes or diabetes of infancy), Maturity onset Diabetes of the young (MODY) and other types of diabetes, such as mitochondrial diabetes (e.g., maternally inherited diabetes with deafness) comes under the category of monogenic diabetes (Radha and Mohan, 2017; Hattersley et al., 2009; Firdous et al., 2018; Rubio-Cabezas et al., 2014). Figure 1 explains the different modes of monogenic forms of diabetes. The story of NDM begins with the newly born infants do not produce enough insulin and this leads to elevated glucose levels (Mohora and Stoicescu, 2016). MODY genes, on other hand is impacted in both adolescence and adults. Diabetic mutations can also be found in mitochondrial DNA which are maternally inherited (Misra and Owen, 2018). Maternally inherited diabetes and deafness from mitochondrial DNA mutations are affecting around 1% of diabetes patients in position 3243 (m.3243A > G), most typically resulting from a substitution A-G (Goto et al., 1990). Both type 1 diabetes mellitus (T1DM) and type 2 diabetes mellitus (T2DM) are polygenic diabetes, which is associated with multiple genes. Polygenic form of diabetes often runs in families and is diagnosed based on serum glucose levels. However, both T1DM and T2DM have substantial distinctions and significant heterogeneities (Diabetes and Clearinghouse, 2014, Sacks and Mcdonald, 1996). The difference between T1DM and T2DM is that in T1DM, insufficient insulin is produced in the body, whereas inadequate insulin is used in the body in T2DM. In the pancreas, the hormone insulin is produced to contribute to the regulation of blood glucose levels. When insulin fails to react with human organism, it is known as insulin resistance/hyperinsulinemia, which further leads to prediabetes, followed by T2DM and cardiovascular diseases. Prediabetes is defined as the existence of glucose values above the normal and below the abnormal values. Prediabetes and insulin resistance are demonstrated when insulin is not used in the human body (Wen and Yang, 2017). A loss of function mutation in the *INS* gene in heterozygotes leads to familial hyperinsulinemia, which has been found to be associated with diabetes via autosomal dominant inheritance. Later, genetic defects with dominant, recessive, maternal inheritance, or X and Y linked inheritances were found to be related to pediatric (neonatal) and adult diabetes. Additionally, the Mendelian form of diabetes accounts for pediatric diabetes (Barbetti et al., 2018; Groop, 2015). Diabetes develops after the neonatal period, which results from complex interactions between moderately penetrant genetic and environmental factors (Lemelman et al., 2018).

NDM is defined as the occurrence of severe hyperglycemia starting from the first month of life in the neonates (Sethi et al., 2020). The global incidence of NDM ranges between 1:90,000–1:160,000 in live births (Katanic et al., 2017; Rubio-Cabezas and Ellard, 2013; Dahl and Kumar, 2020). NDM was first noticed as the beginning of the 9^th^ century, but diabetes in children was not confirmed as T1DM/insulin dependent diabetes and ketotic due to the absence of clinical features (Aguilar-Bryan and Bryan, 2008). NDM is also defined as the occurrence of persistent hyperglycemia in the initial months of the life and further reflecting a harsh reduction in β-cell function (De Franco and Ellard, 2015; Sood et al., 2017). In the future, infants with diabetes may be prone to increased insulin-resistance (Wirth et al., 2018). The clinical symptoms of NDM includes hyperglycemia, intrauterine growth restriction (IUGR), muscle weakness, developmental delay, learning difficulties, epilepsy, failure to thrive and limited ketoacidosis. It may be challenging to detect neonatal diabetes because of certain factors of hyperglycemia in newborns that render identification difficult. This is valid during preterm or low birth weight in the newborns. Preterm babies also have extremely elevated blood glucose levels. The multiple causes of neonatal hyperglycemia are elevated parental glucose levels, sepsis stress, enhanced stress counter-regulatory hormones, phenytoin or glucocorticoid administration and steroid medications (Pun et al., 2010; Lemelman et al., 2018). A previous study confirmed in their case report that children with established NDM can undergo a molecular diagnosis, which offers a rationale for oral hypoglycemic agents, which can improve the lives of affected infants (Pun et al., 2010). Approximately 80% of children with neonatal diabetes have a genetic mutation. The 22 genes have been implicated to the genetic etiology of NDM, which is associated with inheritance pattern, phenotypic, and clinical characteristics (Table 1). NDM related génes are crucial in the formation and synthesis and secretion of pancreatic β-cells. The most common genetic causes of NDM with normal morphological pancreas are mutations of the 6q24 locus and ATP-dependent potassium channel (K_ATP_) gene mutation(s). The K_ATP_ channel plays a major role when the pancreas β-cell stimulates insulin production in response to glycosis (Beltrand et al., 2020). The K_ATP_ channel is an octamer generated in the subunits of the *KCNJ11* and of the *ABCC8* genes; KIR6.2 and SUR1 ion-channel regulation units (Khan et al., 2015). In a consanguineous population, the most prevalent genetic cause was a homozygous mutation in the *EIF2AK3 gene*, which result in 24% of them were affected with Wolcott-Rallison syndrome (Al-Fadhli et al., 2021). Half of the children with NDM can be treated without insulin using the medication Glibenclamide, which controls the high glucose levels (Sperling, 2005). The alteration in the K_ATP_ channel in NDM patients has been successfully treated with sulfonylurea and also in some cases thiamine is also utilized. Therefore, molecular diagnosis is considered an important aspect that can be applied immediately in the management of NDM patients. A satisfactory glycemic control has been achieved with heterogeneous genetic defects in NDM (Ozsu et al., 2016). Based on phenotypic characteristics, NDM is categorized as transient-NDM (TNDM; OMIM #601410), permanent-NDM (PNDM; OMIM #606176) and syndromic forms (Raghav et al., 2017). PNDM and TNDM cannot be recognized clinically at the time of diagnosis; thus, genetic screening should be implemented (Sahebi et al., 2019). One of the commonalities between PNDM and TNDM is that PNDM is a chronic condition, whereas TNDM disappears during infancy and reappears later in life (Nagashima et al., 2017).

**Table 1:** List of documented genes enrolled for diagnosis in NDM children.

Genes	Type of mutation	Locus	OMIM	Inheritances	Phenotypes of diabetes	Features of NDM	Treatment
KCNJ11	Point mutation	11p15.1	600937	Dominant	PNDM/TNDM	DEND syndrome, development delay, low birth delay, seizures and other neurologic features	Insulin Sulfonylurea
ABCC8	Point mutation	11p15.1	600509	Dominant/Recessive	PNDM/TNDM	Low birth weight and DEND syndrome	Insulin Sulfonylurea
EIF2AK3	Point mutation	2p11.2	604032	Recessive	PNDM	Wolcott-Rallison syndrome	Insulin
SLC19A2	Missense & nonsense	1q23.3	603941	Recessive	PNDM/TNDM	Strokes, thiamine responsive megaloblastic anaemia syndrome and seizures	Insulin and thiamine
WFS1	Point mutation	4p16.1	222300	Recessive	PNDM	Wolfram syndrome	-
INS1	Missense mutation	11p15.5	176730	Dominant/Recessive	PNDM/TNDM	Low birth weight	Insulin
6q24 (*PLAGL1* &*HYMA1*)	Over expression (Methylation)	6q24	601410 & 603044	Sporadic	TNDM	Low birth weight, umbilical hernia and IUGR	Insulin
GATA6	Point mutation	18q11.1-q11.2	601656	Dominant	PNDM	Cardiac malformation, Gut abnormalities and gall bladder agencies	Insulin
GCK	Point mutation	7p15-p13	138079	Recessive	PNDM	MODY = GCK	Insulin
FOXP3	Missense mutation	Xp11.23-p13.3	300292	X-linked	PNDM	IPEX syndrome	Insulin
GLIS3	Nonsense frameshift	9p24.3-p23	610192	Recessive	PNDM	Polycystic kidneys, Congenital hypothyroidism and congenital glaucoma	Insulin
IER3IP1	Missense mutation	18q21.2	609382	Recessive	PNDM	Epilepsy and microcephaly with simplified gyration	Insulin
MNXI	Missense mutation	7q36.3	142994	Recessive/Sporadic	PNDM	Short stature, neurogenic bladder and development delay	Insulin
NEURODI	Frameshift/Missense	2q32	601724	Recessive	PNDM	Developmental delay, cerebellar hypoplasia and visual impairment	Insulin
NEUROG3	Nonsense mutation	10q21.3	604882	Recessive	PNDM	Malabsorptive Diarrhoea	Insulin
NKX2-2	Missense/nonsense	14q13.3	604612	Recessive	PNDM	Short stature, hearing impairment and development delay	Insulin
PDXI (IPFI)	Missense mutation	13.q12.1	600733	Recessive	PNDM	Pancreatic agencies in some cases	Insulin
RFX6	Missense mutation	6q22.1	612659	Recessive	PNDM	Small bowel atresia and gall bladder hypoplasia	Insulin
STAT3	Missense mutation	17q21.2	102582	Dominant	PNDM	Early onset multiorgan autoimmune disease	-
GATA4	Missense/deletion	8p23.1	600576	Dominant	PNDM/TNDM	Congenital malformation	Insulin
HNF1β	Missense mutation	17q21.3	189907	Dominant	TNDM	Pancreatic hypoplasia and developmental abnormalities	Insulin
SLC2A2	Missense/deletion	3q26.1-q26.3	138160	Recessive	TNDM	Fanconi Bickel Syndrome	Insulin

### 1.1 TNDM and its mechanism

TNDM is a heterogeneous disorder that affects 50–60% of NDM infants. TNDM disappears during the infancy stage and reappears later in life, i.e., adolescence and requires lifelong therapy (Müller, 2021). The prevalence of TNDM incidence ranges between 1:400,000 and 1:500,000 (Priyadarshi et al., 2015). The probable mechanism might be that the pancreatic dysfunction depends on the severity of the β-cell defects (Li et al., 2012). Slow growth will be one of the factors present in the confirmed TNDM neonates, will have thrive in infancy, with hyperglycemia and dehydration. PNDM is less frequent than TNDM and 70% of TNDM cases are caused by chromosomal 6q24 anomalies, and 25% are caused by mutations in the *KCNJ11* and *ABCC8* genes, which either encode subunits of K_ATP_ channels in the pancreatic β-cells (Gunes et al., 2021). Mutations in the *ABCC8* and *INS* genes account for 10–12% of TNDM cases, respectively (Demirbilek et al., 2015). The mechanism of 6q24 triggers TNDM in human beings remains unclear. TNDM is mainly connected to the *HYMA1* and *ZAC1* genes due to active association with parental and maternal allele silencing (Shield, 2002). To date, three types of abnormalities have been established which result in paternal allele over-expression: paternally inherited chromosome 6q24 reproduction, paternal single-parent isodisomy or a methylation defect chromosome 6 (Novak et al., 2020). In general, TNDM is sporadic; however, parental transmission is known in one-third of the tested patients, which is limited where fathers are non-diabetic (Rubio-Cabezas and Ellard, 2013; Polak and Cavé, 2007). A genetic diagnosis is thus essential in TNDM, since the individuals affected can successfully be treated with sulfonylurea rather than subcutaneous insulin (Polak et al., 2020). In TNDM neonates, IUGR is frequent, and less insulin is required than in PNDM infants (Naylor et al., 2011). Neonates born with the combination of abnormalities with 6q24 and IUGR develops severe non-ketotic hyperglycemia during the initial week of life (Temple and Shield, 2002). Uniparental disomy, or maternal hypomethylation is commonly present in sporadic cases. The *KCNJ11* & *ABCC8* mutations which activate K_ATP_ channel genes are present in 20% of TNDM cases (Fu et al., 2019). Clinical studies have indicated that TNDM patients have no islet or diabetes-susceptible HLA haplotypes (Gardner et al., 2000).

### 1.2 PNDM and its mechanism

**Figure 2:**
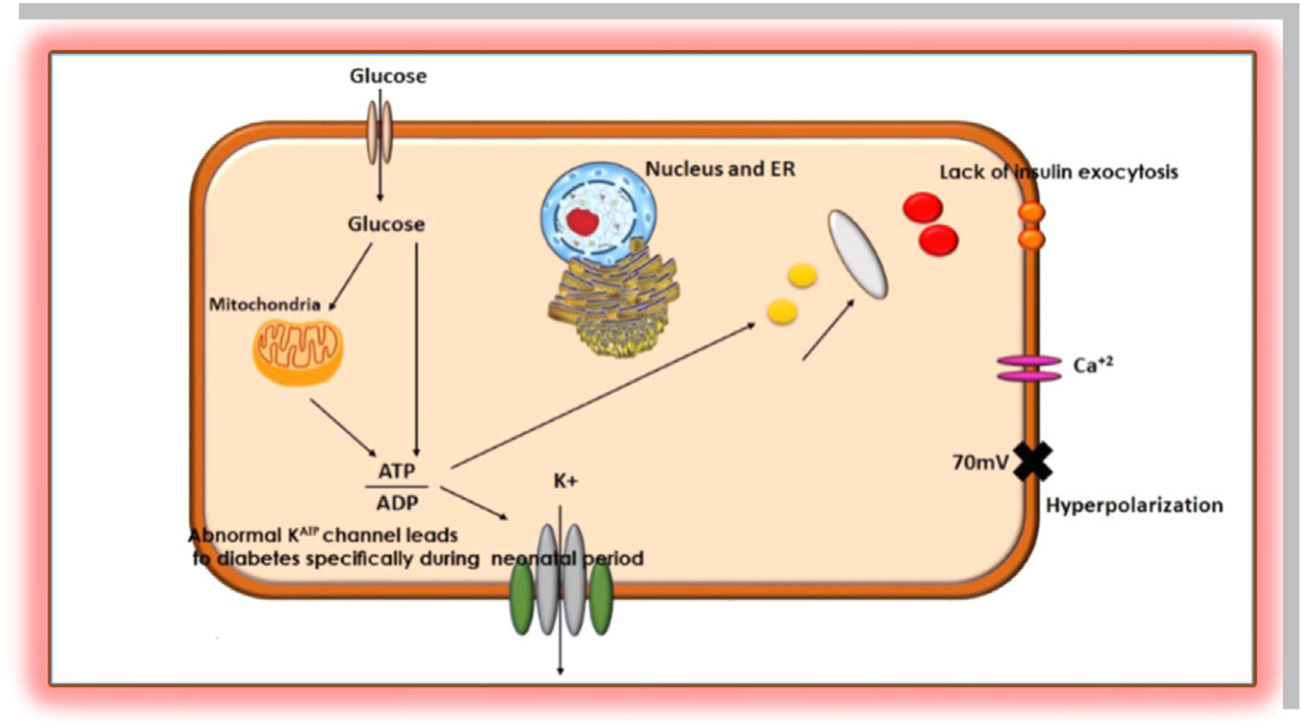
Genetic relation between K^ATP^ channel leads to diabetes; specifically, PNDM.

PNDM is a lifetime alternate type for TNDM which emerges at the beginning of 6 months of life (Ma et al., 2018). Children with PNDM experience slow growth before birth. DEND syndrome was identified as developmental delay in PNDM and early epilepsy, whereas in the first 12 months of a life in term DEND is described as PNDM, with milder developed delays and epilepsies (Taberner et al., 2016). PNDM is one of the rare diseases with the prevalence range of 1:120,000–1:260,000 (Huang et al., 2014). The majority of affected genes in the development of PNDM are *KCNJ11* as an autosomal dominant gene, *ABCC8* and *INS1* as autosomal dominant or recessive genes; *GCK* and *PDX1* as autosomal recessive genes. However, *GCK*, *EIF2AK3*, *FOXP3* and other genes were also involved with the prognosis of PNDM (De León and Stanley, 2016). The deletion and mutation in *PDX1* and *GCK* genes play a major role in pancreatic development and abolishes the enzymatic activity (Metz et al., 2002). The *INS1* gene is the second most common cause after the *KCNJ11* and *ABCC8* genes in the development of PNDM in neonates (Greeley et al., 2011). PNDM is affected by Wolcott-Rallison syndrome and other specific mutations, which are documented in Table 1. Infants suffering from *KCNJ11* and *ABCC8* genetic mutations may benefit from oral sulfonylurea treatment (Huang et al., 2014). A connection was documented between PNDM disease and mutations in the *KCNJ11* and *ABCC8* genes through the Kir6.2 and SUR1 subunits, which inhibit closure of potassium channels and the prevention of insulin secretion and release (Vedovato et al., 2016). The relation between PNDM and K^ATP^ mutations were described in Figure 2.

### 1.3 Discrepancies between T1DM and NDM

NDM is diagnosed only in infants, primarily in the first three months after delivery and treated either insulin or drugs, and varies based on the affected gene, whereas T1DM can develop in all individuals, starting from children to adults; T1DM is caused by the failure of the pancreas to produce insulin. The prevalence of NDM and T1DM varies (Dalwadi et al., 2019). Earlier studies have confirmed that NDM occurs due to reduced birth weight for gestational age (Aguilar-Bryan and Bryan, 2008), while in T1DM, there is no connection with gestational birth weight. A similarity between NDM and T1DM is that both diseases are connected with multiple mechanisms, which can lead to further complications in the future. T1DM is not a rare disease; it can affect any age group, from infants to adults. T1DM develops due to the failure of insulin production in the human body. Insulin is the only treatment for T1DM and there is no single gene defect. In long-term insulin therapy leads to weight gain, obesity, and metabolic syndrome, and drives the expansion of insulin resistance, which further leads to β-cell apoptosis (Wang et al., 2017). NDM is often mistaken as T1DM, which is very rare in a child aged less than six months. However, diabetes diagnosed before six months of age is due to a defect in the gene, implying a genetic cause. NDM occurs as genes deliver the instructions for the production of proteins within cells. If there is an alteration or mutation in the gene, then that protein may not be properly regulated. The cause is genetic mutations that affect proteins that play a key role in the body's ability to produce insulin or in the ability of insulin to lower blood glucose. Individuals typically have a couple of alleles in the gene, inherited from the mother and father (Saraswathi et al., 2019).

### 1.4 Screening of molecular genetic variants identified in NDM

Molecular genetic diagnosis of NDM has helped to confirm the diagnosis, appropriate treatment, and prognostic information of NDM (Attia, 2018). Impaired fasting glucose with diabetes is associated with a single gene mutation in pediatric diabetes. Genetic mutations or silencing of these genes cause diabetes from birth (neonatal diabetes) to childhood, young adulthood (MODY, others), and beyond are *KCNJ11, INS, ABCC8, GATA6, GATA4, RFX6, PDX1, NEUROD1, GCK, NEUROG3*, and *HNF1β* (Barbetti et al., 2018). NDM (PNDM and TNDM) can be inherited genetically from the parents of an infant through autosomal dominant, autosomal recessive, X-linked, and spontaneous mechanisms. Genetic studies have identified the list of genes associated with the development of NDM (Table 1).

### 1.5 Potassium voltage-gated channel subfamily J member 11 (KCNJ11)

The heterozygous gain of functional mutations of *KCNJ11* (OMIM# 600937) are usually dominant and can be either directly decreased by mutation of the ATP bind by residual binding pocket-forming mutations or interfere with the ATP's access to the binding pockets or, while the mutations are far from the bonding pocket of ATP. The K_ATP_ channel is open, and these mutations function by lowering ATP sensitivity, resulting in β-cell hyperpolarization and decreased insulin secretion. DEND syndrome is caused by R201C mutations in the *KCNJ11* gene in one-fifth of the children (Madani et al., 2016). Limited mutations were inherited from an autosomal dominant inheritance that had no imprinting impact, with the bulk of around four-fifth of an emerging de novo. There is a definite relationship between genotype and phenotype for Kir 6.2 mutations (Joshi and Phatarpekar, 2011). Almost 80% of the mutations are denovo, with an autosomal dominant inheritance pattern (Naylor et al., 2011).

### 1.6 ATP binding cassette subfamily C member8 (ABCC8)

The majority of known mutations in the *ABCC8* (OMIM#600509) gene are novel. Both the TNDM and PNDM, or DEND syndrome can result from mutations in *KCNJ11* and *ABCC8* genes. While *KCNJ11* mutations have been the most prevalent, *ABCC8* mutations can be dominant or recessive and are typically inherited. The F132L mutation dramatically reduces the ATP's capacity to block the K_ATP_ channel activity in both homozygous and simulated heterozygous conditions (Proks et al., 2006). The etiology of NDM is based purely on the identification of *KCNJ11* and *ABCC8* mutations (Rubio-Cabezas and Ellard, 2013).The effect of damaging uncommon *ABCC8* mutations on the K_ATP_ channel activity is well documented because of several distinct phenotypes were connected. NDM may be caused by gain-of-function in *ABCC8* mutations and also known as activation, which may occur in newborns (ABCC8-NDM). Recessive or compound heterozygous loss-of-function *ABCC8* mutations, inactivating, causes hyperinsulinemia and hypoglycemia, whereas dominant variants cause hyperinsulinism. A study of people with hyperinsulinism caused by a heterozygous activating inactivating mutation in the *ABCC8* gene, such as p.K1384Q and p.E1506K, discovered that the majority of the patients went on to develop diabetes later in life (Li et al., 2021; Babenko et al., 2006).

### 1.7 Eukaryotic translation initiation factor 2 alpha kinase 3 (EIF2AK3)

EIF2AK3 (OMIM#604032), which causes Wolcott-Rallison syndrome, is another gene typically involved in PNDM. Homozygous mutations in the *EIF2AK3* gene are reported as the most common cause of NDM in individuals born to consanguineous parents (De Franco et al., 2015; Rubio-Cabezas et al., 2009b). Recessive loss of function mutations in the *EIF2AK3* gene reduce the capacity of the endoplasmic reticulum (ER) to deal with stress resulting in loss of functional co-ordination among ER chaperones that govern the synthesis of proteins and aggregation of proinsulin (Fatani, 2019). EIF2AK3 enzyme EIF2A in Ser51 regulates the synthesis of ER unfolded proteins. Targeted EIF2AK3 mice disruption also induces diabetes by the build-up of unfolded proteins that lead to death in the β-cell (Brickwood et al., 2003). A study in the Saudi Arabia has identified the deletion of c.1044-1057del14 in PNDM children (Habeb et al., 2012).

### 1.8 Solute carrier family 19 member 2 (SLC19A2)

The thiamine carrier protein is encoded in the *SLC19A2* (OMIM#603941) gene. Mutations in this gene produce thiamine-responsive megaloblastic anemia syndromes (TRMA) that is an autosomal-recessive condition, characterized by diabetes. TRMA with hyperglycemia and deafness is a thiamine metabolism condition caused by mutations in the thiamine transport gene in *SLC19A2* (Shaw-Smith et al., 2012). Diabetes in a TRMA syndrome is a monogenic form of diabetes owing to *SLC19A2* mutations that encode a thiamine conveyor called THTR1 plasma membrane (Alzahrani et al., 2006). Furthermore, a rare recessive cause of PNDM have been identified, including the *SLC19A2* gene (Labay et al., 1999). So far, 51 missense and nonsense mutations in the *SLC19A2* gene have been identified. Insulin production has been shown to be impaired in the islet cells of thiamine-deficient mice (Demirbilek et al., 2019). In individuals with syndromic diabetes with hearing loss and anaemia, a diagnosis of TRMA should be suspected, even if it is only very moderate and especially with consanguinity (Olsen et al., 2007). c.327_334del, c.428C > T, c.237C > A, c.196G > T and c.602C > T/c are the some of the mutations have been documented in the *SLC19A2* gene (Shaw-Smith et al., 2012).

### 1.9 Wolframin ER transmembrane glycoprotein (WFS1)

In WFS1 (OMIM#222300), the genetic etiology of Wolfram syndrome, NDM was not described in patients with recessive loss of function, even while early start diabetes is a key hallmark of this multi-system disease (De Franco et al., 2017).The genetic contribution of WFS1 in promoting to the risk of diabetes is minimal and can be achieved if an insulin secretion impairment is granted (Florez et al., 2008). Wolfram syndrome is caused by mutations in the *WFS1* gene. It is also known as Diabetes Insipidus, Diabetes Mellitus, Optic Atrophy, and Deafness, a very complex disease, symptoms consisting of diabetes, insipidus diabetes, optic atrophy, surgery, and neurodegeneration. A few children with neonatal-onset insulin-dependence were described to exhibit extra symptoms. Due to the abnormal fold of the protein tungsten, the β-cell degeneration causes endoplasmic stress in multiple tissues. Autosomal recessive mutations may be; nevertheless, several dominant mutations were also characterized (Kocova, 2020).

### 1.10 Insulin (*INS*)

Human *INS* (OMIM#176730) mutations are a common PNDM cause in the neonates has been described as missense mutations (c.184C > T, c.3G > A, -331C > A) in the coding region. Most of the mutations described are expected to disrupt proinsulin molecule folding (Garin et al., 2010). The 646 bp deletion has been detected in two PNDM children from the Lebanon family, comprising exons 1 and 2 of the *INS* gene (Raile et al., 2011). Genetic mutations in the *INS* gene is associated with various forms of NDM. However, precise mutations in the *INS* gene are known as a common cause of NDM. Hyperinsulinemia and late-onset diabetes can result from *INS* gene mutations that produce structurally defective insulins. Autosomal heterozygous mutations are the main cause of PNDM in the *INS* gene causes the ER of the β-cell to misfolding preproinsulin and to build up, resulting in an ER stress and β-cell apoptosis (Yang and Chan, 2016).

### 1.11 6q24

Although most occurrences of TNDM result from chromosome 6q24 (OMIM: 601410 & 603044) abnormalities of the imprinted domain (Temple et al., 2000). *PLAGL1* (ZAC) and *HYMAI* genes are expressed paternally in an impressed area on chromosome 6q24 (Table 2). The most common cause of TNDM generating paternal uniparental disomy is overexpression of these genes due to maternal methylation loss, the contribution of these genes to diabetes is uncertain (Greeley et al., 2010). 6q24-related TNDM must be defined as the TNDM caused by the imprinted locus of genetic abnormality at 6q24 which is determined by using TNDM and DNA methylation analytical testing which demonstrate relative hypomethylation across the 6q24 region. The overexpression of the printed genes at 6q24 is induced by 6q24-TNDM (Temple and Mackay, 2018). 50% of the population with TNDM who test positive for 6q24 develop persistent diabetes.

**Table 2:** Details of 6q24 associated with NDM.

Locus	Genes of Interest	Imprint	Parental origin of imprint	Disease mechanism
6q24	*PLAGL1* and *HYMA1*	Methylated	Maternal	Hypomethylation, paternal UPD or paternal duplication

### 1.12 GATA binding protein 6 (GATA6)

GATA6 (OMIM#601656) variations may be caused by *de novo* mutations in the NDM (Gaisl et al., 2019). The most common cause of NDM due to pancreatic agenesis is heterozygous mutation in the *GATA6* transcription factor gene. PNDM may potentially emerge from pancreatic hypoplasia linked to mutations in pancreatic β-cell developmental transcriptions and *GATA6* is one the most frequent. A novel heterozygous frameshift mutation c.635 660del (p.Pro212fs) was documented (Yau et al., 2017). In NDM subjects, Allen et al. compared neonates with pancreatic agenesis and determined that *GATA6* mutations were present in all of them, implying that almost all patients with pancreatic agenesis had genetic abnormalities in GATA6 (Allen et al., 2012).

### 1.13 Glucokinase (GCK)

Glucokinase (GCK; OMIM# 138079) gene in homozygous and heterozygous compounds mutations, may cause isolated PNDM and are a minority of cases (Massa et al., 2005). This enzyme is crucial in the synthesis of glucose-stimulated insulin, as well as in the absorption and conversion of glucose into glycogen in the liver. Discrepancies are connected with changes in GCK or the function of glucose metabolism and insulin secretion (Mustafa et al., 2019). Homozygous mutations were also found as a rare cause of PNDM for the *GCK* gene (Esquiaveto-Aun et al., 2015). The 8 cases of GCK mutations causing PNDM, includes homozygous and compound heterozygous variants with various types of mutations, all of which result in a complete loss of glucokinase activity. Six of the 8 cases in the GCK-PNDM had been reported in Arabic or European consanguineous families with distant connections between parents. European research of PNDM collections found that full GCK impairment was not a common cause of PNDM, but it should be addressed in families with a history of glucose intolerance or first-degree relatives, especially when consanguinity is suspected (Osbak et al., 2009).

### 1.14 Forkhead box P3 (FOXP3)

The functional purpose of *FOXP3* (OMIM#300292) is the decoding of a fork box transcription factor desired to modulate immunologic tolerance for CD4+ CD25 + regulatory T-cells (Hwang et al., 2018). Immune dysregulation polyendocrinopathy, enteropathy, X-linked (IPEX), also known as X-linked autoimmunity-immunodeficiency syndrome, is caused by mutations in *FOXP3* gene. The loss of these cells therefore leads to an unregulated autoimmune response in men with *FOXP3* homozygous mutations. A study by Rubio-Cabezas et al. has documented the 3 novel mutations with an amendment in the amino-acid substitutions (missense mutations) in the *FOXP3* gene (Rubio-Cabezas et al., 2009a).

### 1.15 GLIS family zinc finger 3 (GLIS3)

NDM and congenital hypothyroidism syndrome is a rare disorder defined by autosomal recessive mutations in the *GLIS3* (OMIM#610192) gene, which collaborates with PDX1, MAFA, and NEUROD1 to regulate insulin gene transcription. Also, in the survival of β-cells and perhaps in insulin separation, GLIS3 is important. GWAS has revealed mutations in the *GLIS3* gene, which has been linked to a wide range of disorders (Splittstoesser et al., 2021; Wen and Yang, 2017). GLIS3 is expressed in the pancreas at various stages of cell development and affects the proliferation of β-cells and the production of insulin (London et al., 2021). Couple of siblings belong to consanguineous family in the Saudi population has been diagnosed with PNDM and other abnormalities such as IUGR, congenital hypothyroidism, hepatic fibrosis, facial anomalies, polycystic kidneys and congenital glaucoma were detected with a nonsense frameshift mutation in the *GLIS3* gene and these siblings has been passed away due to the infection (Senée et al., 2006).

### 1.16 Immediate-early response 3-interacting protein 1 (IER3IPI)

Mutations in the *IER3IP1* (OMIM#609382) gene on chromosome 12 of long arm can cause microcephaly, epilepsy, and diabetes syndrome. In patients with IER3IP1 gene mutations, decrease of activity results in death of neurons and pancreatic β-cells. Molecular investigation has shown that homozygous missense mutation (c.T233C) in the *IER3IP1* gene, a protein that mediate cell differentiation, are an ER stress response protein (Poulton et al., 2011). (Abdel-Salam et al., 2012) reported a similar homozygous mutation (c.T233C) in the *IER3IP1* gene in four children from two unrelated consanguineous Egyptian families, which had previously been documented (Poulton et al., 2011).

### 1.17 Motor neuron and pancreas homeobox 1 (MNX1)

Human PNDM is caused by a homozygous *Mnx1* (OMIM#142994) mutation, with the unknown mechanisms (Pan et al., 2015). MNX1, motor neuron and pancreas encoding Homeobox protein 1, which shows participation in the development of pancreatic and the specification of β-cells in mouse and zebrafish. MNX1 was prominently expressed both in the development and maturity of pancreatic β-cells and in adult pancreatic islets. Bonnefond et al. identified a novel homozygous missense mutation (c.816C > A/p.Phe272Leu) in the second exon of the *MNX1* gene (Bonnefond et al., 2013).

### 1.18 Neuronal differentiation 1 (NEUROD1)

NEUROD1 (OMIM#601724) is a basic helix-loop-helix transcription factor, which is involved in the formation of neuronal components and the endocrine pancreas (Demirbilek et al., 2019). Previous studies were documented with the homozygous frameshift and missense mutations in the *NEUROD1* gene in the PNDM cases. A study conducted by the Demirbilek group discovered a novel missense mutation (p.Ile150Asn(c.449T > A)) in the *NEUROD1* gene within a 13-year-old female child. Parents had the history of second cousin consanguineous marriage with a strong family diabetes paternal history. Both parents and two unaffected male siblings were heterozygous for the mutation (Demirbilek et al., 2019). Additionally, Rubio-Cabezas et al. study discovered a couple of novel homozygous *NEUROD1* mutations in two unrelated probands: a single base pair duplication (c.364dupG) and a two-base pair CT deletion (c.427 428del). Both of these mutations cause a frameshift and a premature truncation of the expressed proteins in C terminus (p.Asp122Glyfs∗12 and p.Leu143Alafs∗55, respectively), resulting in mutant proteins that lack the transactivation domain entirely. There was a history of first-cousin consanguinity between the parents (Rubio-Cabezas et al., 2010).

### 1.19 Neurogenin-3 (NEUROG3)

Studies have identified NEUROG3 (OMIM#604882) to be important in the control of pancreatic development in both mice and humans, acting as a pancreatic islet cell development, pro-endocrine factor in engagement, and pancreatic islet differentiation master regulator. However, *NEUROG3* homozygous loss-of-function mutations have been reported due to abnormal entero-endocrine differentiation, with variable degrees of severity of signs and symptoms and the potential onset of PNDM or pediatric diabetes. Diarrhea is due to congenital malabsorption in the *NEUROG3*. A novel homozygous nonsense mutation p.Q4∗ (c.10C > T, NM 020999.3) in the *NEUROG3* gene was discovered in a male Turkish patient who was born to consanguineous parents and was diagnosed with PNDM and malabsorptive diarrhea with neurointestinal dysplasia (Hancili et al., 2018). Humans with *NEUROG3* homozygous mutations can induce congenital malabsorptive diarrhea and neonatal hyperglycemia. A nonsense mutation (E123X) was discovered in the domain of the *NEUROG3* gene encoding helix II, resulting in premature termination at amino acid 123 (Pinney et al., 2011).

### 1.20 NK2 homeobox 2 (NKX2-2)

Two homozygous nonsense and missense mutations in the *NKX2-2* (OMIM#604612) gene were discovered in three patients from two families, including the p.R129X (c.385C > T) and p.P119fs (c.356 del) mutation. Because they are null, these mutations are highly hazardous. The findings of the Cosegregation study were consistent with recessive inheritance (Flanagan et al., 2014). Human NDM is caused by the *NKX2-2* mutation (Auerbach et al., 2020).

### 1.21 Pancreatic and duodenal homeobox 1 (PDX1)

*PDX1* (OMIM#600733) gene mutations are a rare cause of pancreatic agenesis associated with NDM (De Franco et al., 2013). PDX1 is crucial in pancreatic development and function with homozygous mutations that lead to NDM, IUGR and exocrine pancreas related pancreas agenesis. A couple of novel mutations of Moroccan Caucasian origins were identified in the E178G substitution in the *PDX1* gene (Nicolino et al., 2010). Kulkarni studies validated the discovery of a novel homozygous mutation p.K163R (c.488A > G) in the *PDX1* gene, and parents were found to be heterozygous (Kulkarni et al., 2017). Sanger sequencing analysis revealed biallelic mutations in the *PDX1* gene in 2.9% of PNDM patients. In one of the patients, as well as his affected male sibling, a heterozygous and novel missense mutation (p.A34fsX191; c.98dupC, p.P87L, and c.260C > T) was detected. Both of the other two probands were homozygous for novel *PDX1* mutations (p.A152G; c.455C > G and p.R176Q; c.527G > A). A heterozygous nonsense mutation (p.C18X; c.54C > A) was identified in a fourth PNDM case (De Franco et al., 2013). A novel homozygous mutation (p.Phe167Val) was reported in the region of exon-2 of the *PDX1* gene in an Iranian population (Sahebi et al., 2019).

### 1.22 Regulatory factor X 6 (RFX6)

RFX6 (OMIM#612659) mutations are supposed to cause NDM by the formation of a flawed RFX6 protein in Mitchell-Riley syndrome. NDM was one of the features of this syndrome, and it has been strongly proposed that RFX6 has a specific role in pancreatic formation and β-cell function. A novel mutation on exon 4, c.541C > T, p.R181W in the proband a homozygous missense mutation was detected and both the parents were found to be heterozygous (Zegre Amorim et al., 2015). Concepción et al. investigated *RFX6* mutations in eight (seven probands) children with NDM and other congenital digestive system anomalies (Concepcion et al., 2014). Sansbury et al. studies showed the *RFX6* nonsense mutations c.2176C4T (p.Arg726X) and c.2596C4T (p.Arg866x) which are compound heterozygous for exons 17 and 18, respectively. The fathers had p.Arg726X heterozygous while the mothers had p.Arg866X heterozygous (Sansbury et al., 2015).

### 1.23 Signal transducer and activator of transcription 3 (STAT3)

The active transcription-factor mutations in the STAT3 (OMIM#102582) were associated to early autoimmune disease development and isolated persistent immune mediated NDM. The K392R mutation in the *STAT3* gene was found in PNDM and was associated with the most severe clinical manifestation. It was found in the STAT3 DNA binding domain (Flanagan et al., 2014). However, study results from Saarimaki-Vire Group indicated that the K392R mutation causes a basic developmental abnormality in pancreatic organogenesis in addition to the early first autoimmune (Saarimäki-Vire et al., 2017). It is necessary for the appropriate development and maintenance of the endocrine pancreas that STAT3 signaling occurs in the pancreas. Furthermore, leptin stimulation of STAT3 inhibits insulin production in mice. STAT3 is involved in the regulation of insulin release in basal β-cells, which is linked to diabetes. A novel missense mutation p.P330S in the STAT3 protein binding domain was reported in a 3-month female neonate in the Spain population and the role of P330S mutation leads to lower insulin production in β-cells by improved transcription factor inhibition of Isl-1 (Velayos et al., 2017).

### 1.24 GATA-binding protein 4 (GATA4)

The involvement of the *GATA4* (OMIM#600576) gene in the development of the pancreas has been discovered in mouse models. A *denovo* missense mutation (p.N273K) in the *GATA4* gene and a 8p deletions were discovered in 5 NDM children, and this mutation affects a highly conserved residue in the second finger region of the GATA4 protein (Shaw-Smith et al., 2014). A previous study in Italian male children with NDM, specifically PNDM, found a novel missense mutation (p.Arg319Trp) rather than deletions in the *GATA4* gene, although the absence of diabetes in a couple of heterozygous relatives shows that a dominant-negative effect is improbable (D’amato et al., 2010).

### 1.25 HNF1 homeobox B (HNF1β)

HNF-1β (OMIM#189907) mutations are a rare cause of NDM and endocrine-exocrine diabetes. The *HNF1β* gene is connected to TNDM in neonates, and Ser148Leu heterozygous mutation was documented (Edghill et al., 2006). In an Italian infant, a novel heterozygous nucleotide variant in intron 1 (c.344 + 2 T > C) of the *HNF1β* gene was discovered (Iafusco et al., 2021). A pair of missense mutations (C443G, S148W) in the *HNF1β* gene were discovered in a pair of Japanese siblings, diagnosed with NDM and neonatal polycystic, dysplastic kidneys (Yorifuji et al., 2004).

### 1.26 Solute carrier family 2 member 2 (SLC2A2)

Fanconi Bickel syndrome (FBS) is primarily associated with heterozygous mutations in the *SLC2A2* (OMIM#138160) gene, which encodes GLUT2. FBS is connected with many diseases, one of which is NDM, which is characterized by elevated glucose levels (Greeley et al., 2010). *SLC2A2* mutations are an autosomal recessive cause of NDM that, after common cause elimination, must be considered in consanguineous families or in individuals with TNDM, with or without FBS features. Till now 6 NDM (PNDM and TNDM) cases have been documented with the *SLC2A2* mutations (missense and deletion). Sansbury et al. identified five mutations in five children, three of which are novel missense (p.Ser203Arg, p.Met376Arg, and c.963+1G > A) and two of which are missense (p.Arg53X) and deletion (p.Phe114LeufsX16). The deletion was identified in children with PNDM, while the other four missense mutations were discovered in children with TNDM (Sansbury et al., 2012). The K5X (lysine 5stop) was reported as a novel nonsense mutation in codon 5 (exon-1) of the *GLUT2* gene by sanger sequencing analysis in a female Korean neonate who was diagnosed with NDM and then FBS. This nonsense mutation results in a significantly shortened protein with only 4 of the 524 amino acids (Yoo et al., 2002).

The children with NDM require accurate genetic diagnosis, which is important for correct classification of NDM and for genetic counselling (Støy et al., 2010). Diagnosis of NDM is based primarily on glucose tests and genetic screening was performed with documented variants. Molecular screening in NDM is performed through DNA or Sanger sequencing analysis (Cao et al., 2016). In the past decade, second generation sequencing has been involved in screening patient's DNA to rule out the human diseases for enabling proper diagnosis.

### 1.27 Second generation sequencing techniques

**Figure 3:**
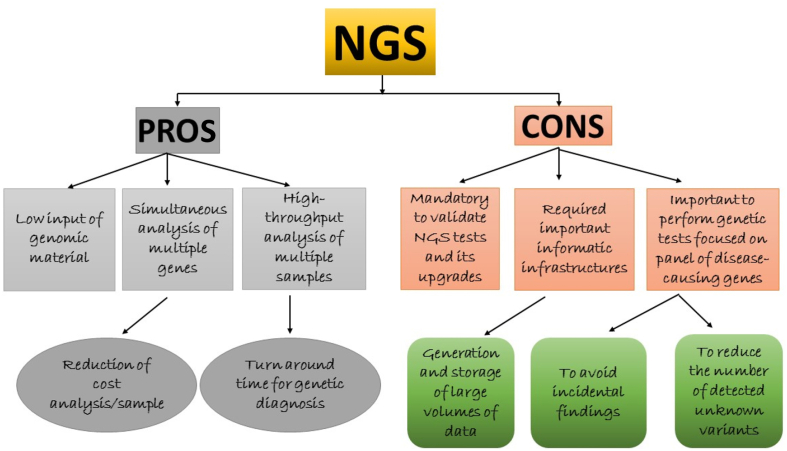
Applications of NGS gene panel testing.

Ultimately, nucleotides (A = T; G≡C) consist of the hereditary information and biochemical properties of telluric life (Heather and Chain, 2016). The nitrogenous bases, sugar, and phosphate groups are connected with nucleotides, and DNA consists of the nucleotide molecules. In each cell, DNA carries the genetic information in a double helical structure. In DNA sequencing techniques, the process determines the nucleotide sequences from limited DNA (Church and Gilbert, 1984). The human genome was successfully sequenced using Sanger sequencing of 3 billion nucleotides of DNA. The obtained genome data were used for the expansion of diagnostic tools, novel therapies, and to predict disease progression (Desai and Jere, 2012). Sanger sequencing was a first-generation sequencing technique, which was introduced by Sanger in 1977 as a commercial sequencing technique, and its application used lower radioactivity and had higher efficiency (Sänger et al., 1977). The Sanger and Maxam-Gilbert techniques were successful in documenting the study of the genetic code in humans (Maxam and Gilbert, 1977). Nominally, sequencing techniques are accurate, quick, cheap, and easily operated. They should also be beneficial for biologists for identifying pathogenic genes, breeding, molecular cloning, and for comparative and evolution studies (Liu et al., 2012). The second-generation sequencing techniques are shotgun sequencing for arbitrary fragmented genomic DNA (FgDNA) or cDNA reverse-transcribed from RNA is performed without the need for cloning by the foreign host cell. Adapter sequences are ligated to either FgDNA or cDNA for the construction of template libraries. Beads on a solid-surface are used for accomplishing amplification of the library. NGS is a powerful platform that has enabled the sequencing of thousands to millions of DNA molecules simultaneously. This powerful NGS method revolutionizes areas such as precision medicine, hereditary disorders and clinical diagnosis by providing a high throughput capacity for many individuals at the same time. NGS technique or high throughput sequencing techniques, such as exome sequencing (ES)/whole exome sequencing (WES) and whole genome sequencing (WGS) are well-known second-generation sequencing techniques, which have been applied by genomic laboratories for molecular genetics. NGS is denoted as a non-sanger, high throughput DNA sequencing technique, which generates millions and billions of small short reads in parallel with the required decreased time compared to the Sanger sequencing technique (Kulski and Challenges, 2016). NGS has started to substitute for outdated molecular techniques, including diagnosing genetic diseases and disorders (De Vries et al., 2005). The advantage of NGS is that complete genome sequencing will take place in less than 24 h; large-scale approaches will increase the speed and lower the cost of DNA sequencing. Another advantage is non-cloning for bacteria because the existence of adapter sequences assists the molecules, which are amplified by PCR (Mostafa et al., 2015). NGS can deliver a clinical diagnosis in a shorter-time and its progress is connected with associated genes in an inherited human disorder to explicate molecular analysis of complex human diseases. NGS is widely used in diagnostics and this technique is required for invasive prenatal diagnosis, infectious diseases, somatic cancers, as well as immune and human hereditary disorders (Figure 3) (Di Resta et al., 2018). The NGS technique randomly sequences the DNA template in the entire genome, which is fragmented to small pieces of DNA and further ligated to designed adapters for arbitrary reads at DNA synthesis (Zhang et al., 2011). In NGS, a huge set of genes and gene panels can be screened in a single test, as a replacement for gene-by-gene screening. The NGS technique is used in clinical genetic diagnostics and research includes common diseases with genome wide association studies (Vrijenhoek et al., 2015). NGS is considerably advanced compared to Sanger sequencing, as it immobilizes solid-phase, multiplexed DNA-fragments enabling array sequencing and *in vitro* clonal constructions of DNA fragments and sequencing libraries (Cao et al., 2017). Presently, NGS has improved in producing more fragments compared with previously, i.e., generating 35–500 bp of short sequences by immobilizing millions of amplified DNA fragments. Thus, it facilitates *de novo*/ES, resequencing, metagenomic studies, methylation and transcriptome profiling (Desai and Jere, 2012).

### 1.28 Exome sequencing

**Figure 4:**
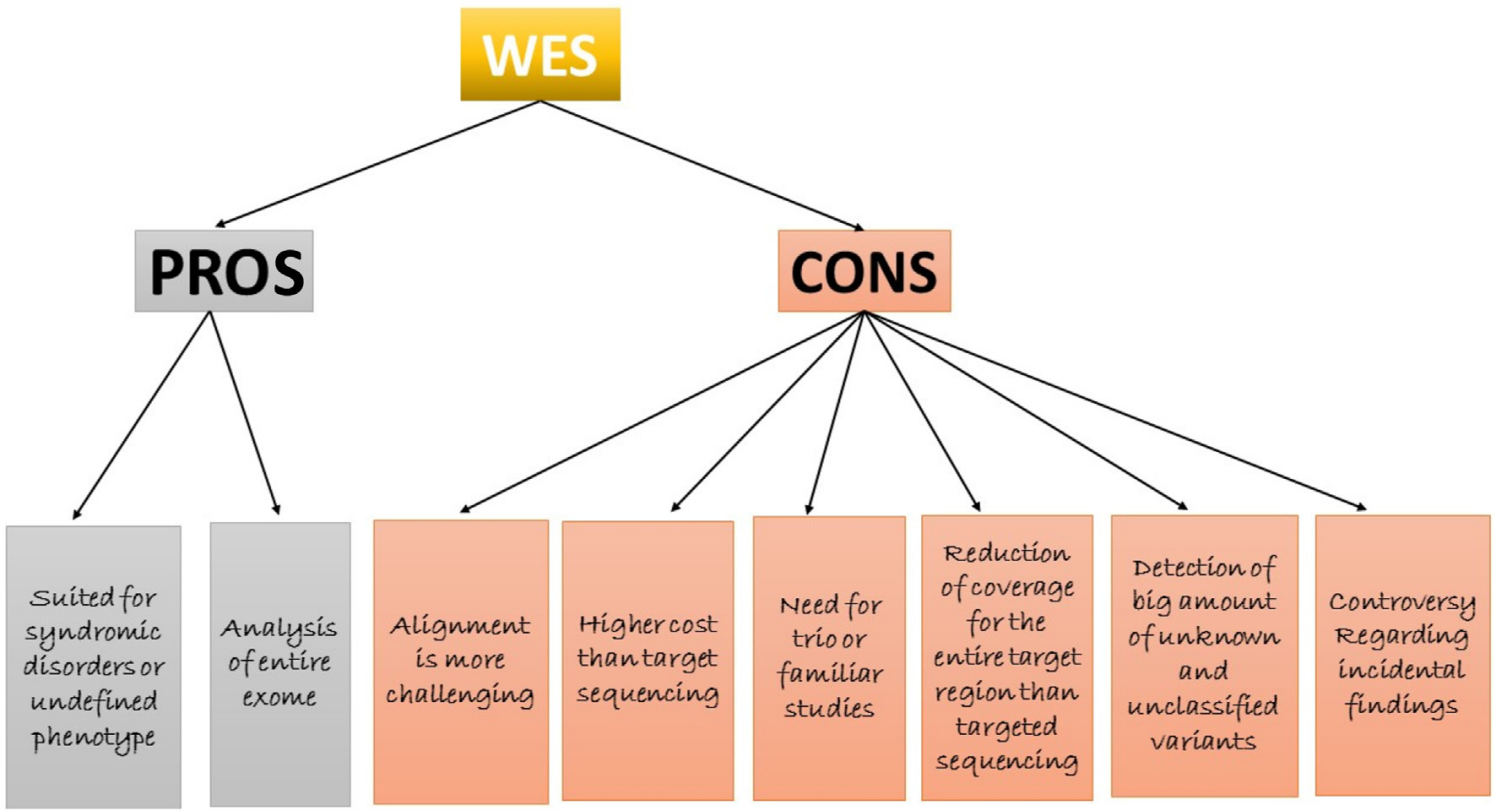
Model for WES screening in human subjects.

ES encompasses the sequence of complete exons in protein coding genes of the genome, depending on the species, which covers approximately 1–2% of the genome. Further, it may extend toward target functional non-protein coding elements and precise candidate loci. Solution- and array-based are two exome capturing techniques that are used (Warr et al., 2015). ES or WES is primarily focused on targeted sequences of protein coding regions of genomic DNA and this sequencing has become a new promising tool for gene detection in complex and unsolved Mendelian conditions for precise diagnosis (Yang et al., 2013). WES is one of the applications of NGS, which is used for perceiving variants. WES captures the sub-genome, which is directly connected with the genome of coding regions; thus, by applying the selection of target and enrichment approaches, the protein-coding region of the genome is sequenced (Cai and Buxbaum, 2013). WES techniques are widely used in clinical applications; in diagnostic tools, it involves lack of coverage in non-coding regions of the genome and flexible depth in coverage of coding regions (Volk et al., 2015). WES provides better clinical and molecular genetic diagnostics compared with NGS and Sanger sequencing because of its cost-effective and high throughput nature involving the single gene approach to gene panels (Mantere et al., 2019). In solution-based WES, the technique is implemented as DNA is further fragmented and probes are applied for hybridization in the target region of the genome. Magnetic beads are used for binding the probes and further amplified by PCR, enriching DNA samples at the target region; in contrast, the array-based WES technique is parallel to solution-based WES, apart from the binding of probes to the high-density microarray (Warr et al., 2015). WES is useful in clinical applications of diagnostics as it completely screens the variations in the exon regions and documents causal variants of a disease or disease-causing mutations. WES has documented the genes connected with Mendelian phenotypes, complex disorders, and Miller syndrome (Suwinski et al., 2019). WES and WGS techniques sequence faster at superior depth, increased sensitivity, and are more feasible (Schwarze et al., 2018). This technique is largely used in the field of molecular genetics and epitomizes a significant breakthrough for diagnostics to detect disease-affecting variants virtually in entire coding regions of the genome (Tetreault et al., 2015). Almost 98% of the ES detects novel and diagnosed variants in the patient's disease precisely. Rare diseases (1:2000) are connected with genetics and adversely affect reproductive fitness. Fifty to seventy-five percent of children are primarily affected with rare diseases. Second generation sequencing techniques (NGS, ES/WES, and WGS) detect each and every exonic region, can also detect the specific pathways in the human genome, and will identify the disease variants (Figure 4) (Wright et al., 2018). However, in rare cases, somatic variants in ES can be missed in disease patients with mosaicism, which exist in the coding region, but are not properly noticed. Numerous variants in cases are responsible for pathology in excess of a couple of genes and structural variants that were not accurately spotted by ES, which is not noticeable in an exon. For specific exome negative cases, there is no proper diagnosis; there are other approaches after the ES, but opting for further steps is experimentally difficult and remains a major challenge (Frésard and Montgomery, 2018). The advantage of WES is that the exome size is moderately small, i.e., 1% of the genome size, and it distinguishes both genetic and nucleotide variants, as well as insertions and deletions in protein coding regions. In non-protein coding regions, the genetic variants such as gene expression regulatory regions will not be detected; this is known to be a major limitation (Sastre, 2014). Both NGS and WES are crucial for molecular characterization of NDM, which should characterize commonly occurring variants for diagnosing the disease in infants or neonates. Second generation techniques will benefit the latest therapeutic agents for treating other forms of diabetes. Specifically, these techniques are beneficial for the diagnosis of NDM with unknown genetic origin, which is required for responding to sulphonylurea (Glyburide), insulin therapy for PNDM neonates, and for future therapy cases (Barbetti et al., 2018).

### 1.29 Comparison between first- and second-generation sequencing analysis in NDM

**Figure 5:**
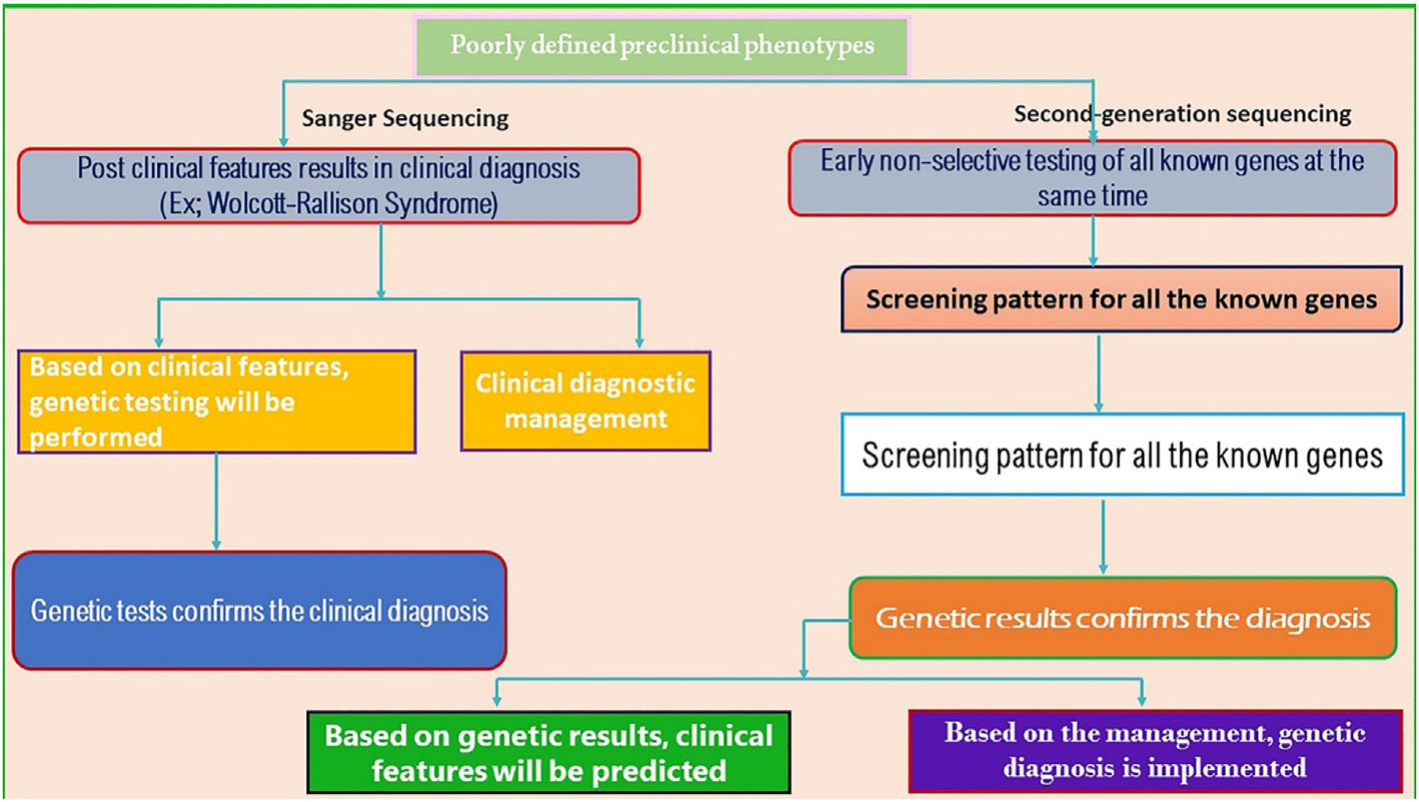
Comparison between sanger sequencing and second-generation sequencing techniques used for the treatment in the NDM children.

To date, 22 known genetic causes of NDM have been reported, including mutations in 21 genes and methylation abnormalities at the 6q24 region (Table 1). DNA-Sanger sequencing allows limited nucleotides to be sequenced in a single reaction, while NGS allows sequencing of the complete genome (_~_19,000 genes) or exome in a single reaction with various times. ES has demonstrated a great potential for documenting disease variants in monogenic disorders. The emergence of NGS could reverse the genetic test methodology, because all genes known for their genetically diverse disorders are analyzed in exome sequencing, as well as customized gene-panel tests for specific disorders. Figure 5 depicts the distinctions between sanger sequencing and NGS techniques used to treat NDM and documents the importance of second-generation sequencing techniques and its uses in treating the NDM neonates for the diagnostic purposes (De Franco et al., 2015). Among them, WES technique is confirmed as a powerful tool to execute genetic research and to identify novel causative mutations, not definitely as a cost-effectively used molecular diagnostic tool (Alrashed, 2014). To date, 100 genes have been confirmed in Mendelian disorders by ES, specifically the *GATA6* and *STAT3* genes in NDM (De Franco and Ellard, 2015; Johansson et al., 2011). Targeted gene panels lower the incident findings for large sequencing depths compared with both WES and WGS, at a lower cost, but do not permit the identification of novel genes (Al-Khawaga et al., 2019). There are limited ES studies that have been documented in NDM in the global population. A study carried out by Cao et al. compared molecular analysis by microarray, Sanger sequencing, and NGS in 18 PNDM and 7 TNDM neonates. The study results confirm six K_ATP_ mutations in PNDM and two *ABCC8* mutations in TNDM, where etiology was reported to be multi-genic inheritance (Cao et al., 2016). Another study by Beryozkin et al. carried-out ES analysis for inherited retinal diseases in 90 patients from 68 families from a combined Israeli and Palestinian population and documents 33 variants which were not reported earlier. Sanger sequencing was additionally implemented to validate the potential pathogenic variants (Beryozkin et al., 2015). A study by Glotov et al. selected 60 unrelated children diagnosed with non-T1DM under 18 years of age in a Russian population for screening, using WES and a panel of 35 genes known to cause PNDM, TNDM, and MODY. WES detected 38 genetic variants and confirmed 27 MODY-related genes, 6 MODY-unrelated genes, 1 variant in *GATA6*, 3 variants in *WFS1*, 1 variant in the *EIF2AK3* gene in only 33 children (Glotov et al., 2019). Al-Khawaga et al. (2019) performed WGS in 9 neonate subjects in the Qatari population and documented commonly occurring deletion variants in the *PTF1A* gene. Using WES analysis, a novel missense mutation in the binding domain of the STAT3 protein was discovered after NDM diagnosis at three months of age in a Spanish population. This novel variant results in aberrant activation of STAT3 and further leads to deleterious downstream effects in pancreatic β-cells (Velayos et al., 2017). A previous study by Bonnefond et al. performed a WES study in a single PNDM French patient and confirmed variants in the *KCNJ11*, *ABCC8*, *INS*, *GCK*, *GLIS3*, *EIF2AK3*, *PDX1*, *PTF1A*, *SLC2A2*, *HNF1B*, and *FOXP3* genes. Additionally, a novel denovo c.1455G > C mutation in the *ABCC8* gene was documented using WES (Bonnefond et al., 2010).

NGS studies were conducted on 24 NDM children from the Oman population, and genetic abnormalities were discovered including *KCNJ11* mutations and 6q24 methylation abnormalities using methylation specific PCR and polymorphic loci, as well as mutations in the *GCK*, *LRBA*, *SLC2A2*, and *IL2RA* genes in 62.5% of the patients (Al Senani et al., 2018). Alkorta-Aranburu et al. utilized NGS and methylation-specific multiplex ligation-dependent probe amplification techniques to study 22 NDM patients and discovered that 36% of TNDM patients had methylation loss and 64% of NDM patients had an unknown cause and for NGS, a panel of 11 NDM genes was screened (Alkorta-Aranburu et al., 2016).

### 1.30 Strength and limitations of second-generation sequencing

Novel mutations are reported in established genes that better comprehend β-cell pathophysiology. The combination of NDM disease with NGS methods as targeted NGS, WES and WGS has proven to be extremely important useful in diagnostic, therapeutic and translational research environments. The strength of second-generation sequencing techniques could be useful in treating the therapeutically targeted by oral medicines in NDM children, resulting to a shift from insulin to sulfonylureas (Nicolaides et al., 2020). Additionally, second-generation sequencing techniques have been applied in human diseases for genetic testing. The advantage of second-generation sequencing techniques is the potential to find causal variants along with familial, novel, and *de novo* variants in the humans. NGS is also adopted for a clear understanding of gene transcription molecules and an unparalleled investigation of the human genome. It also allows numerous genes to be analyzed concurrently for an economical price and will be an alternative to conventional genetic screening (Johansson et al., 2012; Szopa et al., 2015). The advantage of WES is that no pre-selection of genes is required as it permits genetic screening that is not suspected to be allied with a disorder. Finally, although WES represents a powerful tool for genetic diagnosis, in some diseases and disorders it might not be the best approach for providing complete clinical suggestions (Harding and Robertson, 2014).

In addition to their strengths, these techniques also have limitations. There are limited NGS and ES studies documented in the global population with NDM. However, all these results have documented 22 variants that are primarily connected with NDM (PNDM or TNDM) and additionally registered the novel variants. Some of the studies have not documented the panel of NDM genes and this could be due to the selection of the gene panel, patients could be mosaic for NDM, errors in sequencing analysis, improper analysis of the data, infrequent clinical data, contamination or low-yield of genomic DNA, and ethnicity. False negative errors might be one of the utmost important factors for the wrong analysis of human and rare diseases, with a low percentage of cells intended for extremely highly sensitive detection of variants. There are no reports that have documented errors in NDM disease, with either NGS or ES. Another limitation with second-generation sequencing is that it is more expensive than sanger sequencing. Despite the fact that genetic testing is expensive, it is clearly cost-effective, especially in neonatal diabetes, because the proportion of patients who are managed properly is very high. Since WES is high-throughput technology, one of the limitations can affect the patient's diagnosis. The coverage of the target sequences (sensitivity rate) will not be 100%, and additionally, false-negative results might occur owing to the lack of coverage in specific genes.

The highest prevalence rate of NDM worldwide is documented in the Saudi population with a prevalence rate of 1:21,000, followed by the United Arab Emirates with a prevalence rate of 1:31,000 (Habeb et al., 2012, 2020). The accurate analysis for diagnosis will allow correct prognosis and better-informed discussions about the desirability of life-prolonging treatments. The application of NGS is highly recommended in pediatric patients because many genetic ailments have poor prognosis and children may or may not live until adulthood (Thiffault and Lantos, 2016). Confirming the common cause of variants for NDM through second generation sequencing methodology could help in filtering present and future diagnostic slants toward improving the molecular medicine of genetic diagnosis.

## 2 Conclusion

This review concludes that second generation sequencing techniques, specifically WES, have identified similar mutations and genes confirmed in NDM (Bonnefond et al., 2010), while other ES studies have documented and identified additional genetic variants in NDM disease. Documented ES variations should be investigated in other global populations in NDM for reconfirmation.

## Declarations

### Author contribution statement

All authors listed have significantly contributed to the development and the writing of this article.

### Funding statement

This research did not receive any specific grant from funding agencies in the public, commercial, or not-for-profit sectors.

### Data availability statement

No data was used for the research described in the article.

### Declaration of interests statement

The authors declare no conflict of interest.

### Additional information

No additional information is available for this paper.
